# Effects of Fermented Pomegranate Peel Polyphenols on the Growth Performance, Immune Response, Hepatopancreatic Health, and Disease Resistance in White Shrimp (*Litopenaeus vannamei*)

**DOI:** 10.1155/anu/9966772

**Published:** 2024-11-27

**Authors:** Zhoulin Yu, Guangye Liu, Sijie Li, Yucong Hong, Shuyan Zhao, Meng Zhou, Xiaohong Tan

**Affiliations:** ^1^Innovative Institute of Animal Healthy Breeding, College of Animal Sciences and Technology, Zhongkai University of Agriculture and Engineering, Guangzhou, China; ^2^Guangdong Provincial Key Laboratory of Aquatic Larvae Feed, Guangdong Yuequn Biotechnology Co. Ltd., Jieyang, China

**Keywords:** disease resistance, fermented pomegranate peel polyphenols, hepatopancreas health, immunity, *Litopenaeus vannamei*

## Abstract

This study evaluated the growth performance, immune response, hepatopancreatic health, and disease resistance in *Litopenaeus vannamei* fed diets supplemented with fermented pomegranate peel polyphenols (FPPP) for 45 days. Five diets were formulated to contain various levels of FPPP: FP0 (no FPPP), FPPP inclusion at 0.015% (FP1), 0.030% (FP2), 0.060% (FP3), and 0.120% (FP4). The results indicated there were no significant variations in weight gain rate (WGR), specific growth rate (SGR), and feed conversion rate (FCR) of shrimp in all treatment groups (*p* > 0.05), but the survival (SR) of shrimp was significantly higher in all groups with the addition of FPPP (*p* < 0.05). Compared with FP0 group, the contents of total protein (TP) and globulin (GLB) in serum biochemical indexes of FP3 and FP4 groups were significantly increased, and the content of blood urea nitrogen (BUN) was significantly decreased (*p* < 0.05). Compared with FP0 group, the activities of superoxide dismutase (SOD), catalase (CAT), alkaline phosphatase (AKP), acid phosphatase (ACP), and lysozyme (LZM) in the hepatopancreas and serum of FP3 and FP4 groups were significantly increased (*p* < 0.05). Similarly, the activities of glutathione peroxidase (GSH-Px), total antioxidant capacity (T-AOC), and phenoloxidase (PO) in the hepatopancreas and serum of FP2 group were significantly higher than those of FP0 group (*p* < 0.05). In addition, the content of malondialdehyde (MDA) in the hepatopancreas and serum of shrimp in FPPP-added groups was decreased (*p* < 0.05). Compared with FP0 group, the expression levels of SOD, CAT, glutathione S-transferase (GST), LZM, prophenoloxidase (ProPO), penaeidin-3 (Pen3), Crustin, immune deficiency (Imd), Toll, and Relish genes were significantly upregulated in the hepatopancreas of shrimp in FP3 and FP4 groups (*p* < 0.05). Additionally, increasing the addition level of FPPP resulted in a more compact hepatosomal arrangement of the shrimp's hepatopancreas, a more visible star-shaped lumen structure, and a significantly higher number of B cells. Finally, the cumulative SR of shrimp in FPPP groups was significantly higher than that in FP0 group after 7 days of infection with *Vibrio alginolyticus* (*p* < 0.05). In summary, dietary supplementation of FPPP can improve SR, immunity, and hepatopancreatic health and resistance to *Vibrio alginolyticus* of *L. vannamei*.

## 1. Introduction

The white shrimp (*Litopenaeus vannamei*) is a significant euryhaline species in global fisheries and aquaculture, with origins in Ecuador and the Pacific coastal waters of Central and South America [1, 2]. Especially in China, according to the statistics of China Fishery Statistical Yearbook 2023, the mariculture production of *L. vannamei* in China in 2022 is as high as 1.34 million tons, accounting for 80.65% of the global shrimp production. *L. vannamei* has a series of eminent traits such as high-temperature tolerance, strong disease resistance, low nutritional requirements, and fast growth rate and tasty meat, and it is deeply loved by consumers [3–5]. However, aquaculture farmers' pursuit of high yields raises the risk of disease outbreaks in shrimp intensive system, and the disease problem has become one of the major impediments to shrimp aquaculture development [6, 7]. *Vibrio* disease, especially *Vibrio harveyi*, *Vibrio parahaemolyticus*, and *Vibrio alginolyticus*, is one of the most frequent and hazardous diseases affecting shrimp production [8]. Antibiotics can effectively prevent and control the above diseases, but long-term use will destroy the water environment, make pathogenic bacteria produce drug resistance, and drug residues even endanger human health [9, 10]. Hence, the development of environmentally friendly and safe new immunopotentiators or antibiotic substitutes has become an extremely urgent problem to be tackled in the field of aquaculture.

In recent years, medicinal plants and their extracts such as garlic, allicin, fermented lemon peel, and citrus limon peels essential oil have been extensively investigated to maintain health status of aquatic animals [11, 12]. Previous studies have shown that medicinal plants such as *Lycium barbarum*, lemon, and *Astragalus* are rich in an assortment of active ingredients and nutrients [13]. They have the advantages of natural, effective, inexpensive, nontoxic (or low toxicity), less harmful to aquatic animals and the water environment, and are not easy to generate drug resistance [14, 15]. In addition, the effective components of some medicinal plants such as lentinan, fucoidan, and *Andrographis paniculata* extracts can increase the appetite of animals, promote the metabolism and growth of the body, improve the immunity of aquatic animals, and successfully prevent and manage a variety of disease [16–18]. Thus, they can be used as antibacterial agents, antiviral drugs, and immune enhancers in aquaculture.

Pomegranate (*Punica granatum L*.) is a kind of fruit with high nutritional value, which is mainly distributed in subtropical and temperate regions [19]. In China, pomegranate with a long history of cultivation and rich planting resources is a kind of plants which is available for medicine and food [20]. Pomegranate peel accounts for more than 40% of pomegranate fruit and is typically discarded as a by-product of processing [21]. Nevertheless, pomegranate peel contains a variety of bioactive substances, among which polyphenols are the main phytochemicals in pomegranate peel [22, 23]. Numerous studies have proved that pomegranate peel polyphenols can be used for lowering blood lipid, antioxidation, antitumor, anti-inflammatory, and antibacterial [24–27]. These health benefits are attributed to the presence of polyphenol compounds such as punicalagin, ellagitannins, gallotannins, and anthocyanins in pomegranate peel polyphenols [28, 29]. Moreover, studies have shown that fermentation of plants can downregulate the level of antinutritional factors and promote the nutritional quality and efficacy value of traditional foods [30, 31]. Nonetheless, there is no relevant research report on pomegranate peel polyphenols and fermented pomegranate peel polyphenols (FPPP) in the field of aquaculture at present. Therefore, based on the above background, the purpose of this study was to evaluate the effects of FPPP on the growth performance, serum biochemical indices, immune response, and hepatopancreatic health and disease resistance against *Vibrio alginolyticus* in *L. vannamei*. This study represents the first application of FPPP as feed additives in aquaculture and also provides a reliable basis for the development of functional additives for shrimp.

## 2. Materials and Methods

### 2.1. Experimental Diets

The composition and nutrient levels of experimental diets were shown in Table 1. Fish meal, soybean meal, soybean protein concentrate, peanut meal, and pork powder were used as the primary sources of protein, wheat flour was used as primary carbohydrate source, fish oil and lecithin were used as primary lipid source, and the experimental diets with isoproteic (42.43% ± 0.11%) and isolipidic (8.50% ± 0.08%) were designed. The FPPP (60%) used in this study were purchased from Shaanxi Ciyuan Biotechnology Co., Ltd. Five experimental diets containing 0% (FP0), 0.015% (FP1), 0.030% (FP2), 0.060% (FP3), and 0.120% (FP4) of FPPP were prepared. All raw materials were ground with multifunction pulverizer and sieved through 60-mesh sieve, evenly combined with oil according to the proportion in the formula, and then 50% tap water was added and stirred using a mixer. The raw material mixture was made into pellet feeds with particle sizes of 1.0 and 1.5 mm by F-26 pelletizer (South China University of Technology, Guangzhou, China) and then dried in an oven at 50°C. Finally, each group of dried feed was sealed and placed at −20°C until the feeding trials began. The crude protein, crude fat, moisture, and ash contents of the feeds in each group were also determined according to the method established by the Association of Official Analytical Chemists (AOAC) in 2005 and the steps described by Sun et al. [32].

### 2.2. Feeding Trial

The white shrimp feeding trial was carried out in Shenzhen Base of South China Sea Fisheries Research Institute, Chinese Academy of Fishery Sciences (Shenzhen, China). The animal management procedures followed the guidelines of the Animal Care and Use Committee of Zhongkai University of Agriculture and Engineering (ethical approval number: ZHKUMO-2022-055). Before the start of the formal experiment, the shrimps were fed with basic feed for 2 weeks. A total of 300 healthy shrimps, each weighing 2.92 ± 0.09 g, were randomly distributed among 100-L buckets. The upper diameter, lower diameter, and height of the plastic breeding bucket are 550 mm, 420 mm, and 640 mm respectively. These shrimps were then divided into five groups, with three replicates per group and 20 shrimps in each replicate. The shrimps were fed with diets at 7:00, 13:00, and 19:00, respectively, and the daily scale of feeding was 3%–5% of the body weight of them. The water was changed by one-third every day, and the quantity and weight of dead shrimp and feed consumption were recorded in time, and the impurities in seawater were removed by filter bag before water exchange. During breeding, the culture water was static water and continuously oxygenated. Throughout the whole feeding experiment, the range of water indicators was as follows: temperature 28 ± 2°C, salinity 29−31‰, pH 7.8–8.4, ammonia nitrogen <0.1 mg/L, nitrite <0.1 mg/L, and dissolved oxygen >5 mg/L.

### 2.3. Sample Collection

After 45-day feeding trial, all shrimps were counted and weighed after 24 h of fasting, and the growth performance and survival (SR) of shrimps were calculated. Six shrimps were selected at random from each group, and hemolymph was extracted from the pericardial cavity using a 1-mL syringe and placed in a 2-mL centrifuge tube. The hemolymph samples were stored overnight at 4°C and then centrifuged at 4000 r/min for 10 min in a freezing centrifuge, and the upper layer of serum was taken and stored at −80°C in a refrigerator for serum biochemical and enzyme activity assays. Dissect the shrimp on a sterile tray using sterilized surgical scissors and tweezers after hemolymph collection. A portion of the hepatopancreas of three shrimps were placed in a 2-mL cryogenic storage tube and transferred to a refrigerator at −80°C for subsequent detection of immune enzyme activities. Another portion of the hepatopancreas of three shrimps was preserved in RNA later and placed to a −80°C refrigerator overnight for future detection of related gene expression. The hepatopancreas of the remaining three shrimps was placed in a test tube filled with 4% paraformaldehyde solution and stored at room temperature for histological examination.

### 2.4. Growth Performance

The weight gain rate (WGR), specific growth rate (SGR), feed conversion rate (FCR), and SR of shrimps were calculated by the following formulas:

  $$\begin{array}{c} \text{WGR }\left(\%\right)=100\times \left(\text{final body weight}-\text{initial body weight}\right)/\text{initial body weight}, \end{array}$$

  $$\begin{array}{c} \text{SGR }\left(\%/\text{day}\right)=100\times \left(\ln\;\left(\text{final body weight}\right)\right.-\ln\;\left(\text{initial body weight}\right)/\text{experimental days}, \end{array}$$

  $$\begin{array}{c} \text{FCR }\left(\%\right)=100\times \text{total food intake}/\text{weight gain}, \end{array}$$

  $$\begin{array}{c} \text{SR }\left(\%\right)=100\times \text{ final shrimp count/initial shrimp count}. \end{array}$$

### 2.5. Serum Biochemical Index Analysis

According to the research method of Chen et al. [33], the total protein (TP), albumin (ALB), globulin (GLB), blood urea nitrogen (BUN), glucose (GLU), triglyceride (TG), and total cholesterol (TC) in shrimp serum were quantitatively analyzed by automatic biochemical analyzer (Hitachi Ltd. 7180 Serial, Tokyo, Japan).

### 2.6. Assay of Enzyme Activity

The hepatopancreas was homogenized with 0.9% normal saline at 4°C and centrifuged at 2500 r/min for 10 min before enzyme activity was measured. The concentration of the homogenate supernatant was defined by the weight volume ratio, and then the homogenate supernatant was used for the determination of enzyme activity. According to the research method of Yu et al. [34], the contents of superoxide dismutase (SOD), catalase (CAT), glutathione peroxidase (GSH-Px), total antioxidant capacity (T-AOC), malondialdehyde (MDA), alkaline phosphatase (AKP), acid phosphatase (ACP), phenoloxidase (PO), and lysozyme (LZM) in serum and the hepatopancreas of shrimps were quantitatively determined by commercial detection kit of Nanjing Jiancheng Bioengineering Institute (Nanjing, China).

#### 2.6.1. Assay of SOD Activity

The SOD activity in the hepatopancreas and serum of shrimp was determined by SOD assay kit (water-soluble tetrazolium-1 [WST-1] method). The supernatant of the hepatopancreas homogenate and serum were diluted with normal saline to ensure that the inhibition rate was about 50%. The supernatant or serum was mixed with the prepared reagent and reacted at 37°C for 20 min, and the absorbance was measured at 450 nm.

#### 2.6.2. Assay of CAT Activity

The CAT activity in hepatopancreas and serum of shrimp was determined by CAT assay kit (visible light method). The diluted homogenate supernatant or serum was mixed with reagent 1 and reagent 2, reacted at 37°C for 1 min, and then mixed with reagent 3 and reagent 4. Finally, the absorbance of 200 μL mixture was measured at 405 nm.

#### 2.6.3. Assay of GSH-Px Activity

The GSH-Px activity in the hepatopancreas and serum of shrimp was determined by GSH-Px assay kit (colorimetric method). The supernatant of hepatopancreas homogenate and serum was diluted with normal saline to ensure that the inhibition rate was about 50%. According to the kit instructions, the supernatant or serum was mixed with the configured reagent and reacted, and the absorbance was measured at 412 nm.

#### 2.6.4. Assay of T-AOC

The T-AOC in the hepatopancreas and serum of shrimp was determined by T-AOC assay kit (ABTS method). The diluted homogenate supernatant or serum was mixed with reagent 4 and 2,2′-azinobis-(3-ethylbenzthiazoline-6-sulphonate) (ABTS) working solution. After reacting at room temperature for 6 min, the absorbance was measured at 405 nm.

#### 2.6.5. Assay of MDA Content

The MDA content in the hepatopancreas and serum of shrimp was determined by MDA assay kit (thiobarbituric acid [TBA] method). The diluted homogenate supernatant or serum, reagent 1, reagent 2, and reagent 3 were mixed and heated to 95°C in water for 40 min. The tubes were cooled with running water and centrifuged at 4000 r/min at 4°C for 10 min. Finally, the absorbance of 200 μL supernatant was measured at 532 nm.

#### 2.6.6. Assay of AKP Activity

The AKP activity in the hepatopancreas and serum of shrimp was determined by AKP assay kit (colorimetric method). The diluted homogenate supernatant or serum was fully mixed with reagent 1 and reagent 2 in a 37°C water bath for 15 min. Then reagent 3 was added and immediately mixed. After standing at room temperature for 10 min, the absorbance was measured at 520 nm.

#### 2.6.7. Assay of ACP Activity

The ACP activity in the hepatopancreas and serum of shrimp was determined by ACP assay kit (colorimetric method). The diluted homogenate supernatant or serum was fully mixed with reagent 1 and reagent 2 in a 37°C water bath for 30 min. Then reagent 3 and reagent 4 were added and immediately mixed. After standing at room temperature for 10 min, the absorbance was measured at 520 nm.

#### 2.6.8. Assay of PO Activity

The PO activity in the hepatopancreas and serum of shrimp was determined by PO Elisa kit (competition method). The homogenate supernatant or serum was added to the antibody precoated enzyme wells, and the biotin antigen working solution was added. After incubation at 37°C for 30 min, the wells were washed using phosphate-buffered saline with Tween-20 (PBST). Then horse radish peroxidase (HRP) was added and incubated at 37°C for 30 min. Following incubation, repeat the previous washing process. Then the color reagents A and B were added and mixed well and reacted at 37°C for 10 min in the dark. Finally, the stopping solution was added, and the absorbance at 450 nm was measured.

#### 2.6.9. Assay of LZM Activity

The LZM activity in the hepatopancreas and serum of shrimp was determined by LZM assay kit (turbidimetric method). The diluted homogenate supernatant or serum was mixed with the configured application bacterial solution. After 15 min of water bath at 37°C, remove and place in an ice water bath below 0°C for 3 min. Finally, the mixture was poured into a 1 cm optical diameter cuvette, and the transmittance was adjusted to 100% with double distilled water at 530 nm to determine the transmittance.

### 2.7. RNA Extraction and Gene Expression Analysis

Total RNA was extracted from the hepatopancreas of shrimps by the Animal Total RNA Isolation Kit from Chengdu Fuji Biotechnology Co., Ltd. (Chengdu, China). Then, the purity and concentration of each RNA were detected by NanoDrop One Microvolume UV Spectrophotometer (Thermo Fisher Scientific, USA), and the integrity of total RNA was detected by 1% agarose gel electrophoresis. Finally, the total RNA (1000 ng) extracted in the previous step was reverse transcribed using Evo M-MLV Mix Kit with gDNA Clean for qPCR from Accurate Biotechnology (Hunan) Co., Ltd. (Changsha, China). The complementary DNA (cDNA) template of the product obtained by reverse transcription was stored in a refrigerator at −20°C for subsequent fluorescence quantitative polymerase chain reaction (qPCR).

The primers were designed according to the published articles [35–37] and listed in Table 2. Real-time fluorescence qPCR was performed using CFX Connect Real-Time PCR Detection Systems (Bio-Rad, California, USA) with Taq Pro Universal SYBR qPCR Master Mix Kit (Vazyme Biotech Co., Ltd., Nanjing, China) to detect the expression levels of immune genes in shrimps. The qPCR reaction system and conditions were operated in accordance with the product instructions. Ultimately, the relative expression of each gene mRNA was analyzed by 2^−ΔΔCt^ method with β-actin as the internal reference.

### 2.8. Hepatopancreas and Intestine Histology Analysis

Based on the method of Li et al. [38], the hepatopancreas and intestine tissues of shrimps fixed in 4% paraformaldehyde were dehydrated with different concentrations of alcohol and embedded in paraffin. Subsequently, the paraffin samples were sliced and stained with hematoxylin and eosin (H&E). Finally, the image acquisition software (ImageView, 4.7.14479) was used to observe and take pictures under the microscope (Leica DM500, Germany).

### 2.9. Challenge Test

Once the samples were collected, the remaining shrimps were fed with the original grouped feed for 5 days and were subsequently challenged with *Vibrio alginolyticus*. The *V. alginolyticus* strain ZK2406 (GenBank accession number PP905135) used in the challenge test was provided by the Innovative Institute of Animal Healthy Breeding of Zhongkai University of Agriculture and Engineering, and the steps of the challenge test refer to the method of Zhang et al. [39]. A total of 24 shrimps were chosen at random from each group, and each group was divided into three replicates (eight shrimps per replicate) for challenge. Fifty microliters of *V. alginolyticus* (6.0 × 10^8^ colony forming unit [CFU]/mL) was injected into the muscle of the third abdominal segment of shrimp. The daily management and feeding conditions of shrimp during the challenge were the same as those in the feeding experiment, and the number of shrimps within 7 days of challenge was recorded, and the cumulative SR was calculated.

### 2.10. Statistical Analysis

All the data were tested for normality and homogeneity of variance, followed by one-way analysis of variance and Duncan's multiple range test. *p* < 0.05 suggested significant difference. Results were shown as means and standard deviations (*n* = 3), and Statistical Package for the Social Sciences (SPSS) 26.0 (IBM, USA) was used for statistical analysis.

## 3. Results

### 3.1. Growth Performance of Shrimps

Table 3 showed the effects of dietary FPPP on the growth performance of *L. vannamei*. There were no significant variations in WGR, SGR, and FCR among all groups (*p* > 0.05). The SR of FP1 (88.33%), FP2 (88.33%), FP3 (96.67%), and FP4 (91.67%) groups was significantly higher than that of FP0 (71.67%) (*p* < 0.05).

### 3.2. Serum Biochemical Index

Effects of dietary FPPP on serum biochemical index of *L. vannamei* were shown in Table 4. Compared with FP0 group, the contents of TP and GLB in FP1, FP2, FP3, and FP4 groups were significantly increased (*p* < 0.05). In addition, BUN levels were significantly lower in the FP3 and FP4 groups compared with the other groups (*p* < 0.05). However, there were no significant differences in the contents of ALB, GLU, TG, and TC in each group (*p* > 0.05).

### 3.3. Activities of Antioxidant Capacity and Immune Enzyme of Serum and the Hepatopancreas in Shrimps

**Figure 1:**
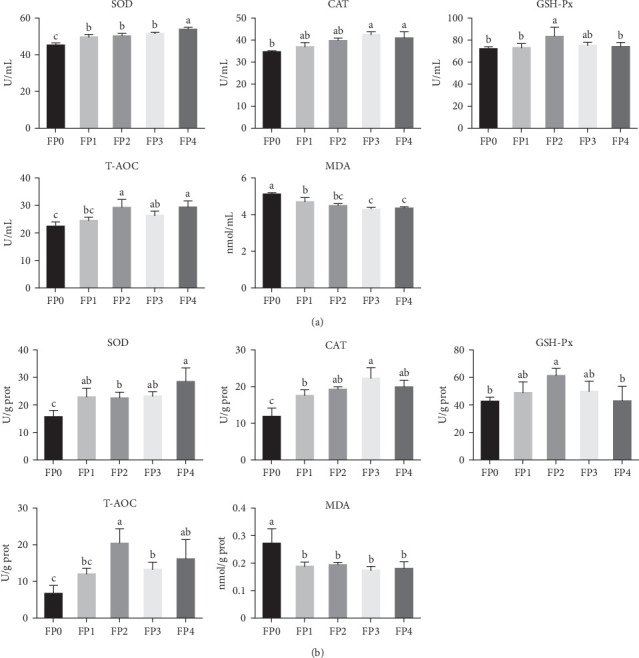
Effects of dietary fermented pomegranate peel polyphenols (FPPP) on superoxide dismutase (SOD), catalase (CAT), glutathione peroxidase (GSH-Px), total antioxidant capacity (T-AOC) activities, and malondialdehyde (MDA) content in serum (A) and the hepatopancreas (B) of *L. vannamei*. Vertical bars represent the mean ± standard deviation (SD) (*N* = 3), and the different letters (a, b, c, etc.) indicated significant differences (*p* < 0.05, analysis of variance [ANOVA]).

Effects of dietary FPPP on antioxidant capacity indexes of *L. vannamei* were shown in Figure 1. SOD, CAT, and T-AOC activity in the FP2, FP3, and FP4 groups was significantly higher than that in FP0 (*p* < 0.05). Meanwhile, the content of SOD in shrimp serum and the content of CAT in shrimp hepatopancreas were significantly higher than those in FP0 group (*p* < 0.05). In addition, GSH-Px activity in serum and hepatopancreas of shrimps in FP2 group was significantly higher than that in FP0 and FP4 groups (*p* < 0.05). On the contrary, compared with FP0 group, the MDA content of FP1, FP2, FP3, and FP4 groups was significantly diminished (*p* < 0.05).

**Figure 2:**
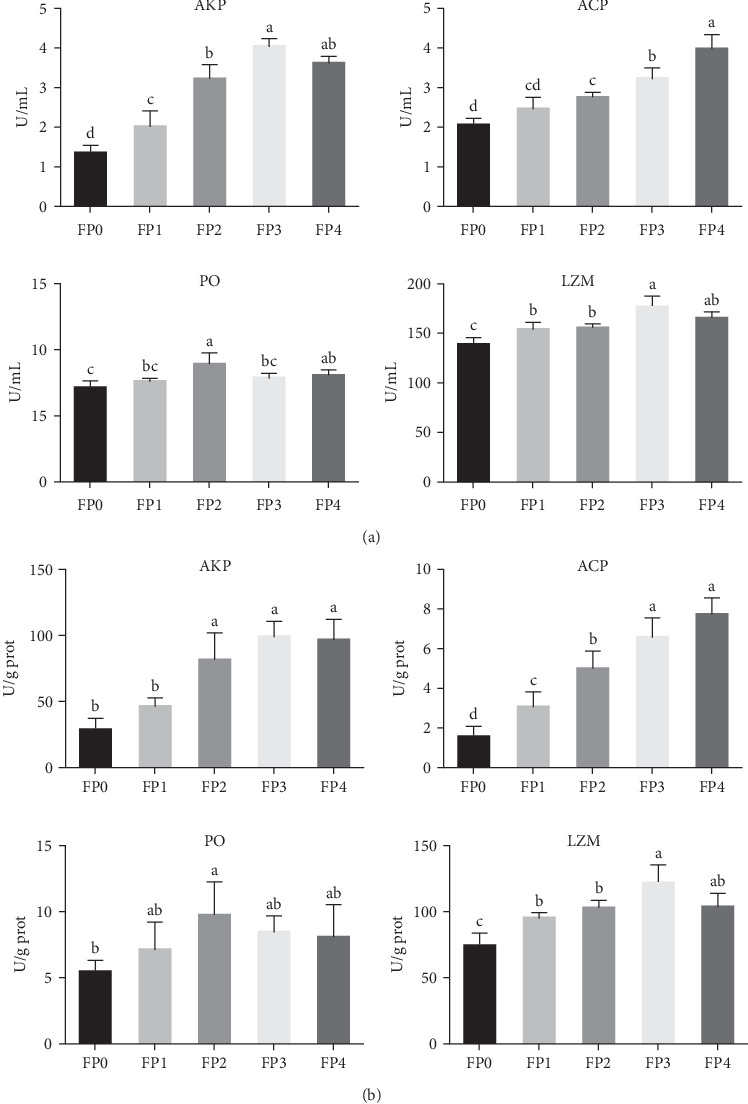
Effects of dietary fermented pomegranate peel polyphenols (FPPP) on alkaline phosphatase (AKP), acid phosphatase (ACP), phenoloxidase (PO), and lysozyme (LZM) activities in serum (A) and the hepatopancreas (B) of *L. vannamei*. Vertical bars represent the mean ± standard deviation (SD) (*N* = 3), and the different letters (a, b, c, etc.) indicated significant differences (*p* < 0.05, analysis of variance [ANOVA]).

Effects of dietary FPPP on nonspecific immune indexes of *L. vannamei* were shown in Figure 2. Compared with FP0 group, the activities of AKP, ACP, and LZM in serum and the hepatopancreas of shrimps in FP2, FP3, and FP4 groups were significantly enhanced (*p* < 0.05). Meanwhile, AKP and LZM contents in serum and AKP, ACP, and LZM contents in hepatopancreas of shrimps in FP1 group were also significantly higher than those in FP0 group (*p* < 0.05). Moreover, PO activity in serum and the hepatopancreas of shrimps in FP2 group was significantly greater than that in FP0 group (*p* < 0.05).

### 3.4. Gene Expression

#### 3.4.1. Expression Levels of SOD, CAT, GSH-Px, and Glutathione S-Transferase (GST) in Shrimps

**Figure 3:**
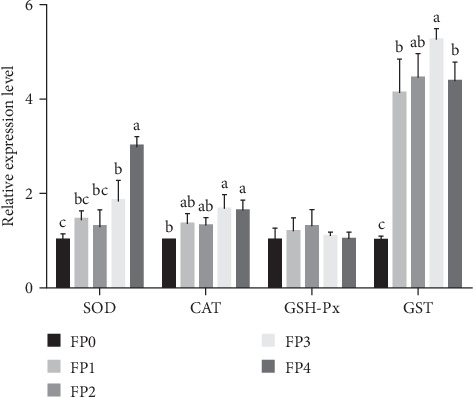
The relative expression levels of superoxide dismutase (SOD), catalase (CAT), glutathione peroxidase (GSH-Px), and glutathione S-transferase (GST) genes in the hepatopancreas of *L. vannamei*. Vertical bars represent the mean ± standard deviation (SD) (*N* = 3), and the different letters (a, b, c, etc.) indicated significant differences (*p* < 0.05, analysis of variance [ANOVA]).

As shown in Figure 3, compared with FP0 group, the expression of SOD and CAT in FP3 and FP4 groups was significantly upregulated (*p* < 0.05). Additionally, the expression of GST in FP1, FP2, FP3, and FP4 groups was significantly higher than that in FP0 group, and the relative expression of FP3 group was the highest (*p* < 0.05). Nevertheless, there was no significant difference in the expression of GSH-Px among all groups (*p* > 0.05).

#### 3.4.2. Expression Levels of LZM, Prophenoloxidase (ProPO), Penaeidin-3 (Pen3), and Crustin in Shrimps

**Figure 4:**
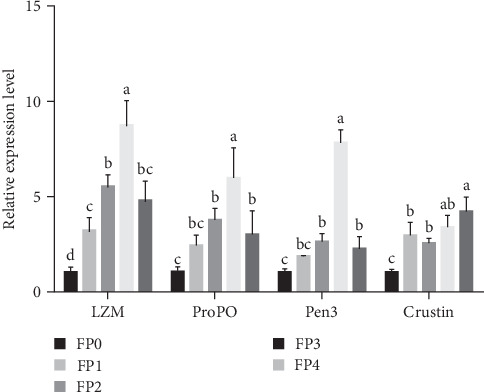
The relative expression levels of lysozyme (LZM), prophenoloxidase (ProPO), penaeidin-3 (Pen3), and Crustin genes in the hepatopancreas of *L. vannamei*. Vertical bars represent the mean ± standard deviation (SD) (*N* = 3), and the different letters (a, b, c, etc.) indicated significant differences (*p* < 0.05, analysis of variance [ANOVA]).

As specified in Figure 4, compared with FP0 group, the expression level of LZM gene in FP1, FP2, FP3, and FP4 groups was significantly increased, and FP3 group was the highest (*p* < 0.05). Additionally, the expression levels of ProPO, Pen3, and Crustin genes in FP2, FP3, and FP4 groups were significantly higher than those in FP0 group (*p* < 0.05).

#### 3.4.3. Expression Levels of Immune Deficiency (Imd), Toll, and Relish in Shrimps

**Figure 5:**
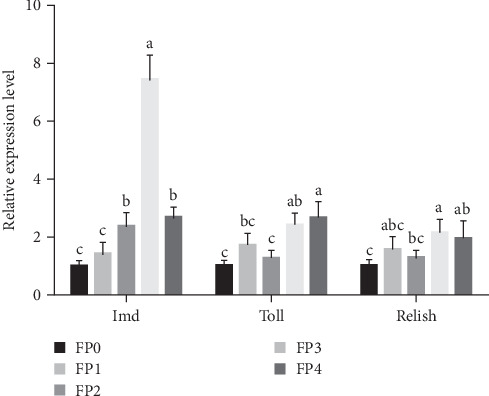
The relative expression levels of immune deficiency (Imd), Toll, and Relish genes in the hepatopancreas of *L. vannamei*. Vertical bars represent the mean ± standard deviation (SD) (*N* = 3), and the different letters (a, b, c, etc.) indicated significant differences (*p* < 0.05, analysis of variance [ANOVA]).

As presented in Figure 5, compared with FP0 group, the expression level of Imd gene increased substantially among treatment groups except for FP1 group, and FP3 group was the highest (*p* < 0.05). Furthermore, the expression levels of Toll and Relish genes in FP3 and FP4 groups were significantly greater than those in FP0 group (*p* < 0.05).

### 3.5. Hepatopancreas Histology

**Figure 6:**
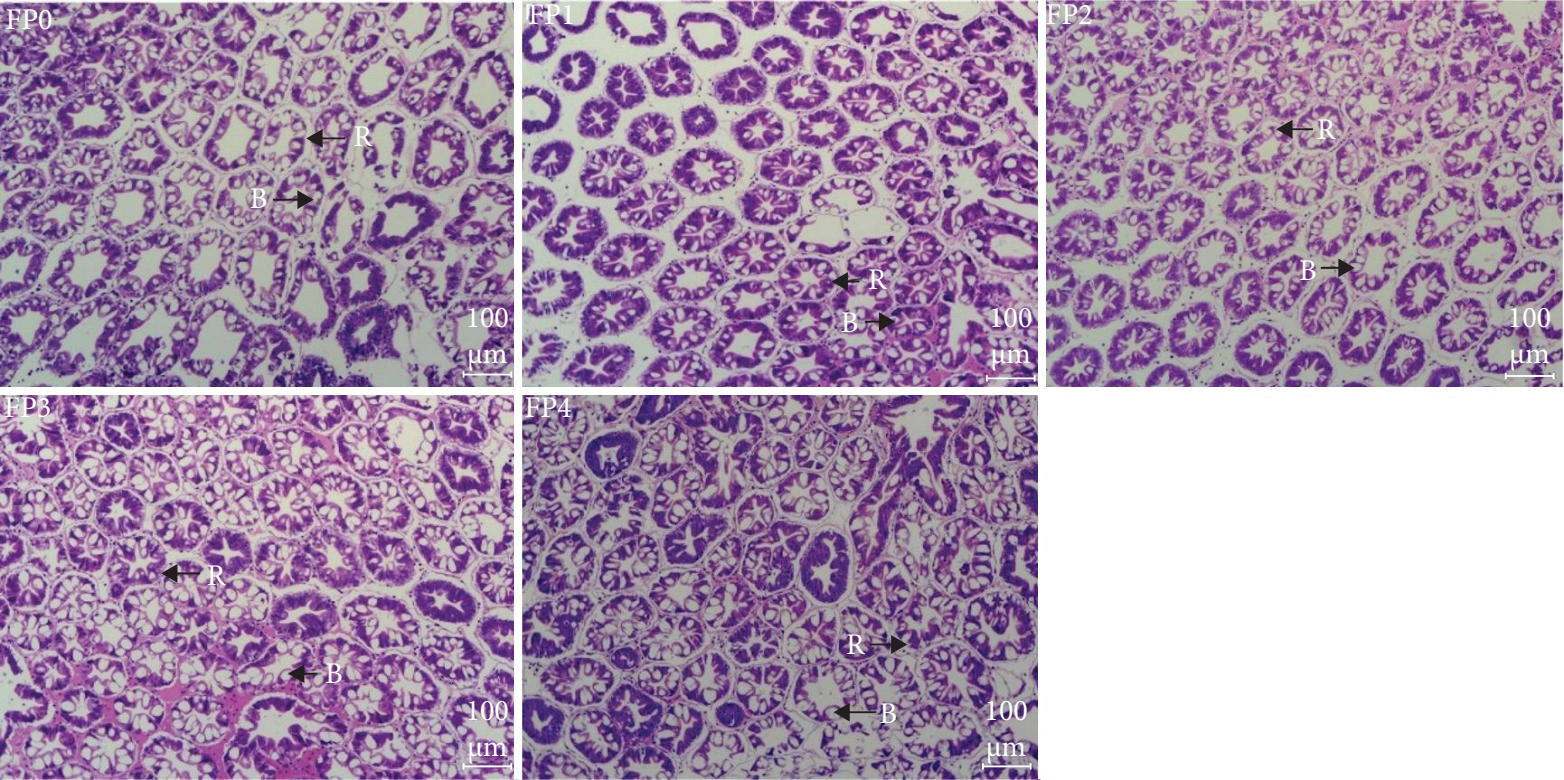
Effects of dietary fermented pomegranate peel polyphenols (FPPP) on the hematoxylin and eosin (H&E)-stained sections of the hepatopancreas of *L. vannamei* (letters in the figure indicate B, B cells, and R, R cells). Magnification ×100.

The effects of dietary FPPP on hepatopancreas histology of *L. vannamei* were shown in Figure 6. In FP0 group, the liver corpuscles of *L. vannamei* exhibited a loose arrangement, with some showing basement membrane shedding and a loss of the star-shaped lumen structure. Additionally, the B cells in the hepatopancreas of the FP0 group were damaged, and their numbers were sparse. However, with the increase of FP addition, the arrangement of liver corpuscles was more compact, and the star-shaped lumen structure was more obvious, and the B cells were further increased, and the number was greatly increased.

### 3.6. Challenge Test of *Vibrio alginolyticus*

**Figure 7:**
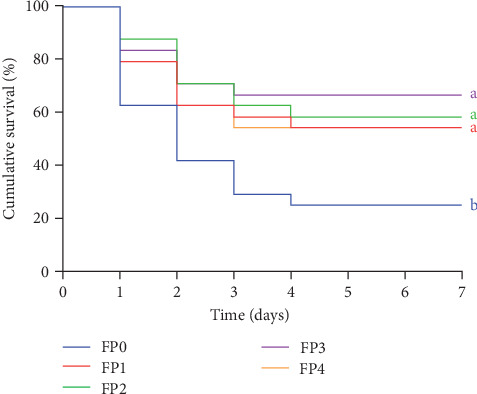
Effects of dietary fermented pomegranate peel polyphenols (FPPP) on cumulative survival of *L. vannamei* after challenge with *V. alginolyticus*. Different letters indicate significant difference (*p* < 0.05, analysis of variance [ANOVA]).

Effects of dietary FPPP on the cumulative SR of *L. vannamei* infected with *Vibrio alginolyticus* within 7 days was presented in Figure 7. The shrimps in each group mainly died within 1–4 days after injection of *Vibrio alginolyticus* and tended to be stable and no longer died after 4–7 days. The cumulative SR of FP1, FP2, FP3, and FP4 groups was significantly higher than that of FP0 after 7 days of challenge (*p* < 0.05).

## 4. Discussion

Serum biochemical indexes are closely related to the metabolism of living organisms and are commonly used to determine the nutritional and health status of aquatic animals, as well as their ability to adapt to environmental stresses [40, 41]. In the present study, the addition of FPPP to the feed increased the serum levels of TP and GLB and decreased BUN of *L. vannamei*. Proteins in the blood have the role of maintaining osmotic pressure, transport, immunity, repairing tissues, and storing energy. Furthermore, TP in serum is synthesized in the hepatopancreas, which contains ALB and GLB, and the elevated levels of TP and GLB indicate enhanced protein synthesis and immune function of the organism [42]. Similarly, the level of BUN reflects the absorption and metabolism of proteins. When the level of BUN in the serum decreases, it indicates that the protein metabolism and the balance between amino acids in the body are in good condition [43, 44]. By coincidence, it has been found that the addition of probiotic bacilli to the feed can promote the accumulation of proteins in the plasma of shrimps to enhance the body's immunity [45]. Similarly, Geng et al. [46] found that dietary *Ampithoe sp*. meal reduced the level of BUN in the hemolymph of shrimps and reduced the accumulation of ammonia in the organism by promoting the synthesis of glutamine and urea. From the above analyses, it is clear that the addition of FPPP to the feed has a positive effect on protein accumulation and metabolism as well as immunity in *L. vannamei*.

Crustaceans mainly rely on their nonspecific immune system to defend against the invasion of foreign pathogenic microorganisms or environmental stress, and antioxidant defense system plays a vital role in it [47, 48]. SOD, CAT, GSH-Px, and GST are the key enzymes in the antioxidant defense mechanism, which can be involved in free radical scavenging [49–51]. Furthermore, T-AOC is a crucial index of the antioxidant status in cells and MDA as the most important product of lipid peroxides; its content can reveal the degree of free radical attack on cells [52, 53]. In the present research, the addition of various concentrations of FPPP to the feed resulted in a great increase in SOD, CAT, GSH-Px, and T-AOC and an enormous decrease in MDA in serum and the hepatopancreas of *L. vannamei*. Analogous results have been reported in the breeding experiments of other plant extracts. Liu et al. [54] added artemisinin to shrimp fed cottonseed protein concentrate feed which can significantly increase the content of SOD, CAT GSH-Px, and enormously reduce the content of MDA. Coincidentally, Cui et al. [55] also found that *Atractylodis macrocephalae* polysaccharides at a safe dose could significantly elevate the content of T-AOC, SOD, GSH-Px, and CAT in hemolymph and intestine of shrimp. Consequently, the addition of appropriate amounts of FPPP to the feed could reduce the risk of free radical damage and improve the antioxidant capacity of the organism in *L. vannamei*.

In addition to its antioxidant defense system, *L. vannamei* is able to rely on its effective cellular and innate immune response to remove foreign particles and resist pathogenic microorganisms [56, 57]. AKP and ACP are not only indispensable indicators of hepatopancreatic immunocompetence in shrimp but also involved in the hydrolysis and metabolism of the phosphatase group [58, 59]. PO is the terminal enzyme in the ProPO system of crustaceans, which can promote the synthesis of melanin to prevent pathogen invasion and promote phagocytosis [60, 61]. Moreover, LZM is also one of the most crucial immune factors, which can improve the immunity and disease resistance by catalyzing the hydrolysis of bacterial cell wall [62, 63]. In this study, the enzyme activities of AKP, ACP, PO, and LZM in serum and the hepatopancreas of shrimp were detected, and the outcomes showed that the addition of different doses of FPPP all resulted in varying degrees of increase in the levels of the above immune-related enzyme activities of *L. vannamei*. This corresponds to the findings of Luo et al. [64] who found that the addition of different strains of acetic acid bacteria to the feed upregulates the levels of AKP, ACP, LZM, and PO in shrimps. Moreover, shrimp can also rely on innate immunity as a defense against microbial invasion through the activation of various immune genes. qPCR results showed that adding 600 mg/kg (FP3) and 1200 mg/kg (FP4) FPPP to the feed could significantly upregulate the levels of LZM, ProPO, Pen3, Crustin, Imd, Toll, and Relish genes in shrimp. Among them, Pen3 and Crustin can both inhibit the growth of pathogenic microorganisms by regulating phagocytosis of hemocytes as different types of antimicrobial peptides in shrimp [65–67]. Similarly, Imd pathway and Toll pathway, as two important signaling pathways in innate immunity of shrimp, can defend against diseases by controlling different kinds of antimicrobial peptides [68, 69]. Furthermore, Imd, Relish, and Toll are key genes in the above two signaling pathways, and Crustin and Pen3 can also be regulated by both the Toll pathway and the Imd pathway [70, 71]. Zhuang et al. [39] also found that the addition of high doses of bovine lactoferrin could significantly upregulate the expression of signal-related genes such as Toll, Relish, JAK (Janus kinase), and signal transducer and activator transcription (STAT), thereby enhancing the immunity of shrimp. Based on these results, we reasonably speculate that high doses of FPPP can modulate the Imd and Toll signaling pathways as well as increase the activities of immune-related enzymes, thereby enhancing the immunity of *L. vannamei*.

The hepatopancreas is not only the main organ for absorption and metabolism in *L. vannamei* but also the source of immune molecules required to resist pathogens and play an essential part in the immune response [72–74]. There are many kinds of cells in the hepatopancreas of shrimp, R cells are mainly involved in the absorption and storage of nutrients, and B cells are involved in the synthesis and secretion of digestive enzymes [75, 76]. In this study, it was discovered that increasing the addition level of FPPP resulted in a more compact hepatosomal arrangement of the shrimp's hepatopancreas, a more visible star-shaped lumen structure, and a significantly higher number of B cells. This result is analogous to the results of Chen et al. [77] which confirmed that the hepatopancreas basement membrane became intact and B cells and R cells were more abundant with the increase of the dose of trans-cinnamaldehyde in the feed. By coincidence, Ye et al. [78] also found that the addition of moderate doses of melatonin to the feed resulted in tightly aligned shrimp hepatic tubules with intact basement membranes and good cell proportions. Therefore, FPPP not only did not cause damage to the hepatopancreas of shrimp but also improved the health status of the hepatopancreas.

Related studies have confirmed that the SR ability of aquatic animals after infection with pathogenic microorganisms can be used to evaluate their disease resistance and immunity [79]. *Vibrio alginolyticus* is one of the typical *Vibrio* that mainly infect *L. vannamei*, which can infect host cells and affect body growth and metabolism by generating toxin [80, 81]. Nonetheless, medicinal plants have been widely confirmed to be used to enhance the resistance of shrimp to *Vibrio* infection. Huang et al. [82] revealed that the SR of shrimp fed with *Bidens alba* and *Plectranthus amboinicus* extracts was considerably improved after being attacked by *Vibrio alginolyticus*. In addition, Lee et al. [83] also reported that fermented lemon peel as a functional additive can increase the resistance to *Vibrio alginolyticus* by enhancing the nonspecific immune response of *L. vannamei*. In this experiment, the cumulative SR of *L. vannamei* in FPPP-added groups was considerably increased after 7 days of infection with *Vibrio alginolyticus*, and this outcome is also consistent with the foregoing analysis of the enhancement of antioxidant capacity and immunity of shrimps. Thus, it is reasonable to speculate that the increased SR of *L. vannamei* after *Vibrio* infection may be related to the ability of FPPP to enhance their antioxidant capacity and immunity and improve hepatopancreatic health. The above results indicate that FPPP can be used as a new type of immunopotentiator to improve the ability of shrimps to resist foreign bacteria.

## 5. Conclusion

In summary, the addition of FPPP to the feed can significantly improve the antioxidant capacity and immunity of shrimp and improve their hepatopancreatic health. In particular, dietary FPPP were effective immunostimulants to control *V. alginolyticus* infection. According to the results of this study, the recommended addition dose of FPPP (60% purity) for *L. vannamei* was suggested to be 0.06%–0.12%.

## Figures and Tables

**Table 1:** Composition and nutrient levels of experimental diets.

Diets	FP0	FP1	FP2	FP3	FP4
Ingredients (%)					
Fish meal	15	15	15	15	15
Soybean meal	15	15	15	15	15
Soy protein concentrate	10	10	10	10	10
Peanut meal	7	7	7	7	7
Pork powder	9	9	9	9	9
Wheat flour	24	24	24	24	24
Corn protein flour	5	5	5	5	5
Beer yeast powder	5	5	5	5	5
Fish oil	1.5	1.5	1.5	1.5	1.5
Lecithin	1.5	1.5	1.5	1.5	1.5
Vitamin and mineral premix^a^	2	2	2	2	2
Choline chloride	0.5	0.5	0.5	0.5	0.5
Monocalcium phosphate	1.5	1.5	1.5	1.5	1.5
Microcrystalline cellulose	3	2.985	2.970	2.940	2.880
Fermented pomegranate peel polyphenols	0	0.015	0.030	0.060	0.120
Nutrient levels (%)					
Crude protein	42.39	42.51	42.56	42.36	42.42
Crude lipid	8.49	8.51	8.48	8.40	8.61
Moisture	4.82	4.72	4.61	4.82	4.70
Ash	9.33	9.30	9.31	9.35	9.42

^a^Vitamin and mineral premix provided by Kingkey Smart Agriculture Times Co., Ltd., China (mg/kg diet): vitamin A ≥450,000 IU/kg, vitamin B_1_ ≥1000 mg/kg, vitamin B_2_ ≥1000 mg/kg, vitamin B_6_ ≥1500 mg/kg, vitamin B_12_ ≥5 mg/kg, vitamin K_3_ ≥800 mg/kg, inositol ≥12,000 mg/kg, D-pantothenic acid ≥3500 mg/kg, nicotinic acid ≥2000 mg/kg, folic acid ≥500 mg/kg, D-biotin ≥5 mg/kg, vitamin D_3_ 300,000–400,000 IU/kg, vitamin E ≥8,000 IU/kg, Na_2_SeO_3_ (1%) 20 mg, CuSO_4_ · 5H_2_O (25%) 24 mg, FeSO_4_ · H_2_O (30%) 266.65 mg, ZnSO_4_ · H_2_O (34.50%) 100 mg, MnSO_4_ · H_2_O (31.80%) 120 mg, Ca (IO_3_)_2_ (5%) 50 mg, CoSO_4_·7H_2_O (5%) 10 mg, Mg 20 g, zeolite power 4380.55 mg.

**Table 2:** Sequences of the primers used in real-time qPCR.

Primer name	Primer sequence (5′−3′)	Product size (bp)	Primer efficiencies	Source
*β-Actin*	F: GAGCAACACGGAGTTCGTTGT R: CATCACCAACTGGGACGACATGGA	68	0.96	[35]
*SOD*	F: AGCCAATGACGTAAGCG R: ACCATCACAAGAAACCC	107	0.92	[35]
*CAT*	F: AGAGGGTTGTGCATGCTAAG R: CAGCTGATCCACTCTCACCT	159	0.92	[37]
*GPx*	F: TTTTTCCGTGCAAAAAGGAC R: TAATACGCGATGCCCCTAAC	242	1.05	[36]
*GST*	F: CACCTACGAACACTACGAAC R: GGTTCTTGAAGCCGTCGAG	128	0.99	[36]
*LZM*	F: TGTTCCGATCTGATGTCC R: GCTGTTGTAAGCCACCC	126	0.96	[35]
*ProPO*	F: TCCATTCCGTCCGTCTG R: GGCTTCGCTCTGGTTAGG	119	1.07	[35]
*Pen3*	F: CACCCTTCGTGAGACCTTTG R: AATATCCCTTTCCCACGTGAC	141	0.93	[36]
*Crustin*	F: GAGGGTCAAGCCTACTGCTG R: ACTTATCGAGGCCAGCACAC	157	0.96	[36]
*Imd*	F: TCACATTGGCCCCGTTATCC R: ATCTCGCGACTGCACTTCAA	117	1.02	[35]
*Toll*	F: TGGACTTCTGCTCGGACAAC R: GTACATGTCCTTGGTCGGCA	116	0.93	[35]
*Relish*	F: CCTGTGAAGACATTAGGAGGAGTA R: CCAGTTGTGGCATTCTTTAGG	208	1.01	[35]

Abbreviations: *CAT*, catalase; *GPx*, glutathione peroxidase; *GST*, glutathione S-transferase; *Imd*, immune deficiency; *LZM*, lysozyme; *Pen3*, penaiedin 3a; *ProPO*, prophenoloxidase; qPCR, quantitative polymerase chain reaction; *SOD*, superoxide dismutase.

**Table 3:** Effects of dietary FPPP on the growth performance of *L. vannamei*.

Diets	FP0	FP1	FP2	FP3	FP4
IBW (g)	2.55 ± 0.15	2.46 ± 0.02	2.60 ± 0.05	2.45 ± 0.04	2.54 ± 0.10
FBW (g)	6.83 ± 0.72	7.20 ± 0.65	7.41 ± 0.18	6.95 ± 0.68	6.76 ± 0.35
WGR (%)	167.19 ± 17.97	192.30 ± 26.49	185.66 ± 3.88	184.21 ± 28.40	166.74 ± 19.11
SGR (%/day)	2.18 ± 0.15	2.38 ± 0.20	2.33 ± 0.03	2.31 ± 0.23	2.18 ± 0.16
FCR	1.73 ± 0.43	1.60 ± 0.15	1.54 ± 0.02	1.64 ± 0.29	1.75 ± 0.18
SR (%)	71.67 ± 15.28^b^	88.33 ± 7.64^a^	88.33 ± 5.77^a^	96.67 ± 2.89^a^	91.67 ± 2.89^a^

*Note:* Values are shown as mean ± standard deviation (SD) (*n* = 3). Means in the same column with different letters (a and b) are significantly different (*P*  < 0.05).

Abbreviations: FBW, final body weight; FCR, feed conversion rate; FPPP, fermented pomegranate peel polyphenols; IBW, initial body weight; SGR, specific growth rate; SR, survival; WGR, weight gain rate.

**Table 4:** Effects of dietary FPPP on serum biochemical indexes of *L. vannamei*.

Diets	FP0	FP1	FP2	FP3	FP4
TP (g/L)	13.70 ± 2.91^c^	18.70 ± 0.26^b^	19.73 ± 1.05^ab^	21.40 ± 1.65^ab^	22.67 ± 1.85^a^
ALB (g/L)	1.30 ± 0.26	1.43 ± 0.06	1.53 ± 0.21	1.37 ± 0.25	1.47 ± 0.21
GLB (g/L)	12.40 ± 2.76^c^	17.27 ± 0.32^b^	18.20 ± 1.11^ab^	20.03 ± 1.46^ab^	21.20 ± 2.05^a^
BUN (mmol/L)	0.30 ± 0.03^a^	0.27 ± 0.05^ab^	0.25 ± 0.04^ab^	0.22 ± 0.02^b^	0.15 ± 0.02^c^
GLU (mmol/L)	0.93 ± 0.15	0.73 ± 0.13	0.85 ± 0.25	1.03 ± 0.26	0.82 ± 0.13
TG (mmol/L)	0.21 ± 0.02	0.23 ± 0.04	0.25 ± 0.03	0.24 ± 0.02	0.24 ± 0.03
TC (mmol/L)	0.20 ± 0.03	0.23 ± 0.03	0.25 ± 0.05	0.21 ± 0.01	0.23 ± 0.03

*Note:* Values are shown as mean ± standard deviation (SD) (*n* = 3). Means in the same column with different letters (a, b, and c) are significantly different (*P*  < 0.05).

Abbreviations: ALB, albumin; BUN, blood urea nitrogen; FPPP, fermented pomegranate peel polyphenols; GLB, globuli; GLU, glucose; TC, total cholesterol; TG, triglyceride; TP, total protein.

## Data Availability

The data that support the findings of this study are available from the corresponding author upon reasonable request.
